# Regulation, modification, and evolution of remote sign language interpreting in Sweden – a service in progress

**DOI:** 10.1186/s12913-024-11907-y

**Published:** 2024-11-19

**Authors:** Camilla Warnicke, Marie Matérne

**Affiliations:** 1 University Health Care Research Center, Faculty of Medicine and Health, Örebro University Hospital, Region Örebro County, Box 1613, Örebro, 701 16 Sweden; 2 School of Behavioural, Social and Legal Sciences, Örebro University, Fakultetsgatan 1, Örebro, 701 82 Sweden

**Keywords:** COVID-19, Interaction, Interpreting, Interpreting agency, Interview, Organisation, Qualitative content analysis, Remote interpreting, Sign language

## Abstract

**Background:**

The sign language interpreting service has undergone a tremendous change due to COVID-19 and remote interpreting has become a more frequent alternative to the face-to-face format. The aim of the study is to investigate how the interpreters perceive the organisation of remote interpreting in Sweden and how it has evolved since the COVID-19 pandemic.

**Method:**

Interviews with 26 experienced remote interpreters, representing 19 of Sweden’s 21 counties, were analysed with qualitative content analysis.

**Results:**

Three themes were revealed in the analysis. The first theme was regulation. It was stated that directives and regulatory decisions concerning provision of remote interpreting services were varied and unclear. Several different platforms were used when interpreting remotely. Some of the services had conducted risk analyses, whereas others had not. The second theme was modification, including adjusting interactions to suit the preferences and capabilities of the users (both signing and speaking parties), as well as adjustments to work environments and workplaces. The third theme, evolution of remote interpreting, showed that support and training were rare and varied. Although the processes and organisation of remote interpreting are not yet fully established in Sweden, remote interpreting is here to stay.

**Conclusions:**

In Sweden, remote interpreting is a service that varies according to regulations and the type of assignments. The service would benefit from being more uniform and streamlined across Sweden, although consideration must be given to those involved with the service.

**Supplementary Information:**

The online version contains supplementary material available at 10.1186/s12913-024-11907-y.

## Background

Sign language interpreting (SLI) became a regular service in Sweden in 1976. In some senses, it is still in its infancy and is undergoing constant evolution and transformation [1]. Due to the COVID-19 pandemic, and because of recent technical developments, opportunities for remote interpreting (RI) have become an option for deaf signers. RI facilitates communication between people who sign (in the case of Sweden, in Swedish sign language, hereafter STS) and people who use a spoken language. An interpreter interprets between them from a studio or on-site at the side of one of the interlocutors.

SLI has undergone a tremendous shift towards RI in several western countries, not only in Sweden but also, for example, in the US, the UK, the Netherlands, Germany, Finland, and Belgium [2]. In 2020, as the risk of transmission of the COVID infection grew [3], interpreting in Sweden began to shift away from face-to-face service and towards RI.

In Sweden, pre-booked RI is funded with government money, and in case of health care via the regional counties. SLI could be procured by private companies. On-demand RI is categorised under phone calls, which are a part of the Swedish Video Relay Service [4] provided directly by the government [5]. The current study focuses on pre-booked RI.

A previous study of RI in Sweden [6] showed that STS/Swedish interpreters reported both positive and negative experiences of working remotely. The interpreters in that study generally considered it easier to work face-to-face since that enabled a closer connection among the interlocutors. Cooperation among the interlocutors and reliable technical devices were sometimes reported as key factors for RI to work well. RI was considered to be a good option for adapting the work to fit the new environment created by the pandemic. The study [6] points out that the rather new kind of interpreting has influenced interpreters’ work. There was such a rapid increase in RI – from almost no remote assignments to exclusively RI during periods of high spread of the COVID-19 pandemic – that there was no time to establish standard RI practices and procedures. Knowledge about the current state of RI organisation can provide guidance for how to enhance, bolster, and advance the service going forward. The aim of the current study is therefore to investigate how the interpreters perceive of the organisation of RI in Sweden and how RI has evolved since the COVID-19 pandemic.

### Evolution of SLI service in Sweden

SLI service in Sweden has evolved over time. SLI began as a pilot project in Sweden in 1968 [7]. In 1976, the SLI service became a regular part of the regional counties’ “assistance compensation” [8]. However, it was not totally clear in which situations deaf people would receive an interpreter, and demand for SLI increased. In 1981, the Handicap Institute (now the Swedish Agency for Participation [MFD]) issued recommendations for a coordinated interpreting service. A so-called disability investigation was launched in 1989 to review the SLI service, which led to an amendment to the Swedish Health and Medical Services Act [9]. The amendment mentioned SLI regarding the right to interpreting services under the auspices of the regional counties. The legislative change came into effect in 1994 [10, 11], stipulating an expanded on-demand SLI service, which it referred to as “interpreting in daily life”. “Interpreting in daily life” was somehow not clearly defined.

The Swedish National Audit Office was tasked with reviewing how the regional counties used the funding of the state subsidy [12], and it identified several shortcomings. The Swedish government issued a directive for a review of the status of STS [13]. A subsequent report on STS [14] revealed, among other things, that the needs for SLI interpretation were not being adequately met. Further, also this report pointed out that the term “interpreting in daily life” was vague. The Swedish government commissioned the National Board of Health and Welfare to investigate how the regional counties were applying the regulations regarding”interpreting in daily life” [15]. The investigation report concluded that the interpreting agencies organisation of interpreting in daily life was not well organised and it was difficult for the users of the service to get interpretation. In addition, it was stated that the regional counties did not yet fully meet the national disability policy objectives.

Consequently, the Swedish government initiated an investigation into the regulation, financing, organisation, and supervision of the SLI service was initiated. This investigation proposed creating a single state agency responsible for the SLI service [16]. RI is mentioned in this document [16] as a service that might be offered if possible and justified considering the individual needs of the primary interpreting user [16]. The document emphasises that significant improvements in technology and interpreting competence would be needed for RI to be a possible alternative to face-to-face interpreting in the future [16]. However, the government rejected the proposals formulated in the document as it would not be possible to implement them due to budgetary limitations. The Swedish government launched a memorandum [17], the result of which emphasis that RI should be offered where deemed appropriate. However, the investigation was criticised and got rejected. Yet another investigation was initiated, which became SOU 2022:11.

Due to the COVID-19 pandemic, the SLI service was forced to adapt to the new circumstances by increasing the number of RI assignments. During the period of increased RI, the report of the latest investigation, SOU 2022:11 [1], was presented. The report clearly emphasised the need for an analysis of a remote SLI function covering all regional counties, stating that remote SLI “is, however, still immature and in the early stages of its development” ([1] p. 350). Thus, the need to take a closer look at how the RI service is organised has been demonstrated within research [6] and in Swedish official documents (see Table 1).

**Table 1:** Overview of significant events in the Swedish organisation of SLI

Year	Event (in selection)
1968	Piloting of interpreting services for deaf people in Sweden [7]
1976	SLI service becomes a regular part of the county counties [8]
1981	The Handicap Institute recommends a coordinated interpreting service for the deaf
1989	The Swedish disability investigation
1994	Amendment to the law in HSL on the right to SLI for expanded interpreting in daily life for childhood deaf, deafblind, adult deaf and hard of hearing [10, 11]
2003	The National Audit Office shows shortcomings in the interpreting business [12]
2006	The National Board of Health and Welfare states that “interpreting in daily life” as too vague [14]
2008	The National Board of Health and Welfare points out that interpreting in daily life is confusing and difficult to understand [15]
2011	Investigation into the regulation, financing, organisation, and supervision of SLI, suggesting a single agency for SLI [16]
2017	Interpreting service in daily life, suggesting RI as a possible alternative to face-to-face SLI [17]
2022	Investigation SOU 2022:11; Action plan for long-term development of one interpreting service for the deaf, hard of hearing and people with deafblindness

## Methods

The aim of the current study is to investigate the interpreters perceive of the organisation of RI in Sweden and how RI has evolved since the COVID-19 pandemic. This was investigated by an explorative qualitative study based on interviews with interpreters experienced in RI.

An e-mail containing information about the study was sent to all regional counties and private interpreting services in Sweden, and the recipients were asked to forward the e-mail to interpreters who might agree to be interviewed. A total of 26 interpreters contacted the first author and indicated that they wanted to participate in the study. All of them were included. The interpreters represented 19 of the 21 regional counties in Sweden. They were employed for both private companies (*n* = 7) and regional counties (*n* = 19) (Table 2). The participants comprised 22 women and 4 men, which reflects the distribution within the profession of SLI. The participants were between 27 and 61 years of age and had been working as interpreters between 1.5 and 38 years.

**Table 2:** Characteristics of the informants

	***n* = 26**
Age, mean (range)	42.7 (27–61)
Sex, n	
Women	22
Men	4
Years in profession, mean (range)	16 (1.5–38)
Employment sector	
Region	19
Private	7
RI experience (assignments per year)	
Much (> 50)	22
Moderate (16–50)	2
Limited (< 16)	1
Not reported	1

The interpreters were interviewed between October 5, 2021, and June 13, 2022. The interview guide was semi-structured and consisted of three sections: 1) the experiences of the interpreters, 2) the organisation of RI, and 3) the interpreters’ teamwork. The first area has been analysed and results have been published [6]. The current study focuses on the second area, the organisation of RI. The interview guide was piloted before starting the interviews, and some formulations were changed to clarify the questions. This pilot interview was not included in the study.

The first author (CW) conducted all the interviews by telephone. She has about 25 years of experience as a certified interpreter between Swedish and STS. Additionally, she is an associate professor, with experience as an interviewer, and researcher.

The interviews were audio recorded and lasted between 60 and 90 min. All the interviews were then transcribed verbatim by a secretary. The chosen method was qualitative content analysis [18]. Both authors thoroughly reviewed the transcriptions to familiarise themselves with the material. NVivo 11 (QSR International, Inc., Cambridge, MA, USA) was used for the analysis.

In the first step of the analysis, the second author (MM) identified significant meaning units that could address the study’s aim; these units were then condensed. The second step was to create codes from the condensed meaning units while still maintaining the content at a manifest level. The codes were subsequently grouped together and organised into categories [19, 20]. The first author then reviewed the categories, and both authors discussed the phrasing until consensus was reached. The final step involved both authors independently creating themes, which sometimes necessitated merging multiple categories. A subsequent discussion ensued. This independent work with the themes and the discussion was a way of seeking trustworthiness of the material [21]. An overarching theme was finally identified to capture the whole data material. The checklist Consolidated Criteria for Reporting Qualitative Research (COREQ; [22]) was used to ensure that all reporting aspects were included in the manuscript. In the results section, the themes and categories are illustrated by quotes from the informants, indicated by a hashtag.

### Ethics approval and consent to participate

The Swedish Ethical Review Authority, a government institution, approved this study (Dnr. 2021–02182). Information about the study (both written and oral) was given to all informants beforehand. All informants gave written informed consent to participate before the interview (see appendix).

## Results

The regional counties differed in how they managed the rapid shift to RI. Analysis of the informants’ perspectives on the organisation of RI yields an overarching theme: RI as a work in progress. This overarching theme is an interpretation of the latent content of the data. Results of the analysis are presented under the three themes of Regulation, Modification, and Evolution. These themes illustrate the organisation of RI in Sweden as it has evolved since the COVID-19 pandemic and will be elaborated on in Sects. "Regulation Modification Evolution". An overview of the results is presented in Table 3 below.

**Table 3:** Overarching theme, themes and categories

**Overarching theme:** RI as a work in progress	
**Themes (3)**	**Categories (7)**
1.Regulation	1.1 Unclear directives and decisions 1.2 A range of platforms are used 1.3 Risk analysis varies
2.Modification	2.1 Adjustments of interaction to suit user preferences and capabilities 2.2 Adjustments to work environments and workplaces
3.Evolution	3.1 Support and training for RI are rare 3.2 RI is a work in progress, but is here to stay

### Regulation

This theme focuses on how RI is regulated in the regional counties around Sweden. It reveals data that is formulated in three categories: 1) Unclear directives and decisions, 2) A range of platforms are used, and 3) Risk analysis varies.

#### Unclear directives and decisions

There are differences among the regional counties in terms of the organisation of RI and regulations governing what interpreters are allowed to do in their professional work. Within regions, directives can vary depending on the type of assignment and whether there is a need to manage the interpreted event at all. For example, in GP-patient consultations, secrecy is prioritised. Some platforms that are used in RI are considered unsuitable for this type of interpreted event, but other (more secure) platforms may have technical limitations that make the interpretation difficult. It is sometimes unclear what to place first: secrecy or usability. Some of the interpreters reported that they solved the problem by choosing a less secure platform to make the interpreting possible. It was obvious that there were unclear directives for some of the interpreters:

*There have been some varying directives, and different interpreters are differently inclined to try to find technical solutions because we were at such different levels from the start, or maybe still are. While some were quite quick to say: yes, but this doesn't work, ... one just found something to solve the situation. (*#14)

Besides the question of how to offer RI, there was also the matter of how to perform the service. The regional counties provided few or no guidelines in this respect. One interpreter described how she tried, in a non-regulative way, to formalise the remote work to some extent to achieve more standardised procedures for RI:

*I have set up guides on how to set everything up ... but not guidelines, no.* (#14)

It is not only the interpreters that are involved in organising and preparing for assignments. Bookings for interpreting assignments are received by a secretary at the interpreter agency. The booking must include enough information to determine whether the assignment can and should be offered remotely or face-to-face. In addition, the interpreter on the assignment needs sufficient information to prepare for it. The staff at the interpreter agency have a significant role, but not all of their tasks are clearly set out:

*Then there’s a lot about how it works with the staff at the interpreter agency, making sure they are also on track with remote interpreting, and when it is suitable and when it is not – that’s another issue. And ensuring that enough information comes through in the order to me as an interpreter, it is not always clearly defined whose responsibility. So, in the beginning, it was often like that, with some users getting frustrated because the coordinating staff called three times, and then two different interpreters called.* (#14)

RI directives are not totally clear, although the interpreters stated that the way bookings are handled has evolved.

The transition from face-to-face to more RI interpreting started abruptly as the pandemic forced a change from interpreting on-site to remote delivery. Due to the pandemic, all assignments became remote, something that was completely new for some of the regional counties:

*It started with Corona. Because we didn’t offer remote interpreting at all before Corona, we had to adapt during the pandemic.* (#12)

By the time we were conducting interviews for this study, outbreaks of COVID-19 were coming and going, and decisions about whether to use face-to-face versus RI were not static:

*Now, during the pandemic, it was a bit different. Just last week, the head of administration came out and said: “We are now only doing interpretation assignments remotely again. We have to decide if there are exceptions because we have an extraordinary situation again.”* (#12)

Governmental as well as organisational guidelines appeared to vary, and decisions were influenced by the pandemic situation. Most of the interpreters stated that neither those who need interpreting nor the interpreters or even interpreter agencies had the final decision on the way interpreting would be offered during lockdown due to COVID-19. The shifting restrictions during the pandemic give the strongest and final judgement. In less risky times, it is the involved parties and the circumstances that determine whether interpretation will be provided remotely:

*First and foremost, it is the technical conditions that determine whether it can be done on-site or remotely. Then it’s the nature of the assignment, what needs to be covered, and how the requester or the primary and secondary interpreter users feel about doing it remotely. We always have a dialogue about what is possible.* (#3)

A first step towards RI is thus to decide whether to carry out the assignment face-to-face or remotely, and after that, to determine which platform to use.

#### A range of platforms are used

As described in the previous section, not all counties have the same regulations setting out which platform to use. At the beginning of the transition towards more RI, some organisations determined which platforms were safe to use. The responsibility for secrecy and safety seemed to vary. One interpreter stated that the user of the service (the signing or the speaking party) was the one who makes the final decision on platform:

*It is the one who book the interpretation that invites – for example a workplace – then they decide what kind of platform to use, we just say thank you and tick [that box] off. Then if we run Teams or Zoom or whatever, […] it’s their responsibility.* (#14)

However, another interpreter reported that the regional county had strong regulations concerning what platform to use, although the regulations were not consistent as they were dependent on other factors:

*We have very clear rules for remote interpreting, which platforms we are allowed to interpret in. Although then it is a question of which platform we from the interpreting service can offer to interpret in when we initiate or send a link. Then we can connect on other people’s platforms if we are the ones invited to their meeting. So then there is, yeah, then we don’t follow... then we follow different rules for testing, you could say, [depending on whether] we are the ones who initiate or if we are the ones who are invited.* (#9)

Some regional counties have their own platforms that they use for healthcare appointments when they are the ones to invite participants. The interpreters mentioned several specific platforms that the regional county councils offered or required them to use; those were Alltid öppet/CMR-links (Stockholm County), Mitt vårdmöte/VMR (Västra Götaland County), Vidicue (Skåne County), and Visiba Care (Örebro County, Västebotten County).

Sometimes the regional counties’ accounts did not work well, and the interpreters seemed to solve this in different ways. It seems that the need to perform the work was prioritised over adhering to whatever rules had been set up:

*When I interpret... well, it’s my private account, but it’s still a neutral one that I don’t use in other contexts. But if I were to interpret via Facetime or via Messenger, XXX Private if you say so, so I think it’s a disadvantage, because then I’m offering my personal account– Then maybe it will happen that you become friends on Facebook or – so yes, you invite [that] and it won’t be professional. So [these less private platforms] have only been used on occasion in emergency situations.* (#21)

Several interpreters stated that they had used their personal devices and accounts for interpreting – often when a sudden problem arose. Issues with devices or connections were sometimes solved by using the interpreter’s or the user’s own mobile phone:

*And we have solved it in all possible ways, sometimes you have brought in a mobile phone and then you have called the hearing person and then you have it on the mobile phone and then you have the other via the computer, you know, they have done everything possible to solve it.* (#2)

The interpreters observed that a variety of platforms were used for RI. Most common were Zoom, Teams, and different versions of Skype. Other general platforms that have been used are FaceTime, FHM, Google Duo, Google Hang Out, Google Meet, Messenger, Telia Ace, VMR, Webex, WhatsApp, and university-specific programs.

Overall, most interpreters considered Zoom to be the most suitable platform for SLI. In Zoom it is possible to pin the signing party’s video so it is not “moving around” (#15), whereas Teams is more difficult to handle:

*In Teams sometimes the image disappears as soon as they change speakers and it is very problematic when you have the screenshot up as well, plus they were lagging. We’ve had problems with logins where you get kicked out of meetings, and yes, there are a lot of headaches when working with Teams. And I’ve also had compatibility problems where I have to start up Teams with a certain internet browser for it to work. And if there has been an update, it also thinks that you should update a lot of other things, so all of a sudden you have a lot of special functions on your computer that you never asked for just because you want to use Teams.* (#15)

Several interpreters reported that having such a wide range of platforms was a big issue. They saw a need for *one* secure national platform that works for SLI; for example, it must include the possibility to pin, to show a PowerPoint and to keep the interpreter’s screen visible at the same time, and it must offer consistent high resolution and stability.

#### Risk analysis varies

Confidentiality and security were matters that some of the regional counties have taken into formal consideration. One way to formally evaluate challenges is to conduct a risk analysis, as some regional counties have done. Evaluation in terms of risk analysis was one way to avoid differences in the RI service according to one interpreter:

*We have evaluated quite carefully what we think has been good and what has not been good and how we should change things so that we do things more uniformly with each other so that everyone does not do things differently when on assignments and so on.* (#17)

According to the interpreters, formal risk analysis has first and foremost focused on the safety of platforms: “*thorough clarification of what constitutes secrecy, i.e. correct in terms of security*” (#12). Steps to protect privacy with RI were taken in both the agency and at home in the interpreters’ private residence, and the employers trusted the interpreters’ assessment of confidentiality during the assignments.

Guaranteeing confidentiality when working from home can be a challenge if other parties also work from home or if family members are home during the assignment. Some counties have agencies that the interpreters may work from:

*It is quite a lot of personal responsibility and that is probably one of the reasons that many people actually go to the interpreting agency and sit in the studios there, because you live in cramped quarters, or you have teenage children who are studying at home during the pandemic and so on. So as for guaranteeing [privacy] – I can’t prove, or my boss hasn’t required me to prove, that I’m maintaining confidentiality. But I think that it is part of the interpreter’s ethics and morals that it also applies in this context. So you sit with a headset and the door closed.* (#14)

One interpreter gave an example of the formalised approach to RI set out in documents from the regional county council:

*No, I have not seen any risk assessment. However, I have seen documents on how to behave as an interpreter, we have been sent, what …, that you must use their company logo, it must say the company’s name ... things like that. But I don’t have a risk assessment*. (#25)

Thus, some regional counties have done risk analyses, and some have not. There is an overall discrepancy among interpreting agencies around Sweden.

### Modification

The theme of modification consists of two categories: 1) Adjustments of interaction to suit user preferences and capabilities, and 2) Adjustments to work environments and workplaces.

#### Adjustments of interaction to suit user preferences and capabilities

RI is adjusted to suit the users’ preferences and capabilities. In some areas, the regional counties have an ongoing discussion with the users and their representatives about whether and how to arrange RI. One regional county had a workshop with representatives from a user council and discussed aspects of RI. In smaller regional counties, it seemed to be easier to have a more direct discussion with the users of the service:

*The larger the region, the less communication you have with your interpreter users. And the smaller the region the better, so that you can be open and honest... In XXX, which is my largest region, I think that no, the communication does not exist. We also see on social media that the interpreter users in XXX are not particularly satisfied. (*#25)

The regional counties had to some extent considered making adjustments to suit RI users’ preferences. On an individual level, any requests the (signing) user might make to the interpreter agency are met to the extent possible. Furthermore, interpreters make their own assessments of whether certain conditions or disabilities might make RI difficult for specific users.

Interpreters’ assessment of additional disabilities or skills a user might have influences whether they consider a remote assignment to be possible. It is easier if the interpreter and users have met before and if they feel comfortable with each other. The interpreter may consider it necessary to make extra adjustments in order to enable some users of the service to attend an assignment remotely:

*And then there are also sometimes certain difficulties, I think if it is, it could be the communication, people who have difficulty understanding what an interpreter is doing at all where you need to be more clear and use more body language, then it becomes a bit flat. So it could be a pure communication aspect that makes you feel that the assignment is not suitable.* (#9)

One disability mentioned by the interpreters was deafblindness, a condition where people have a combined vision and hearing impairment of such severity that it is hard for the impaired senses to compensate for each other [23]. Although people with deafblindness may have some vision left and can see visual sign language, it could be difficult to decode the signing in RI. However, some of the interpreters stated that people with deafblindness can benefit from RI:

*Some deafblind people who see so well that you can interpret remotely, it has been an advantage, because you [the deafblind person] can focus on a person if the lighting conditions and background are right... because then you [the deafblind person] don’t have to sit in a room with bad lighting conditions, bright colours, so it has sometimes been an advantage for some.* (#2)

#### Adjustments to work environments and workplaces

As mentioned earlier, interpreters carried out RI assignments either at the interpreter agency or at home during COVID-19, although within these two options the situation varied. In some services, the interpreters were obliged to work from the agency. One interpreter stated that, in the critical part of the pandemic, the studio had to be big enough to allow enough space between two interpreters working in the same room. Before the breakout of COVID-19, several interpreter agencies had no studio at all, and they had to build one (or several) studios for remote purposes:

*They have rebuilt an entire room, repainted and made draperies and, yes, lamps and yes, tidied up. At the [interpreters] agency there is a real sound-insulated door and everything, so that it adheres to all the rules. But I would argue that what got us going was Covid.* (#21)

The services equipped the studios in different ways. The interpreters described how some RI studios were equipped with ergonomic aspects in mind: a desk that could be raised and lowered so interpreters could choose to stand or sit while working. Standing mats and special ergonomic saddle seats were other adjustments. The interpreters stressed that background screens as well as their clothing were considered with a view to creating contrast. Thus, several adjustments were made to make RI comfortable and suitable for interpreting:

*Yes exactly, we have, it feels like we have done what we can to make it a good one, ... it has happened gradually as you have worked with it, you have noticed the needs.* (#6)

The services also made technical adjustments, such as increasing bandwidth and setting up firewalls. In addition, they updated technical devices, procured new computers and added speaker pucks. For assignments conducted from the interpreters’ home, additional adjustments were made to accommodate RI. Some interpreter services fully equipped their interpreters to provide RI from home:

*[A]t home, each of the interpreters have received a kit with equipment and things to facilitate remote interpreting at home as well.* (#10)

However, not all interpreters were equipped to the same extent by their service agencies.

### Evolution

This third theme consists of two categories: 1) Support and training for RI are rare, and 2) RI is a work in progress, but is here to stay.

#### Support and training for RI are rare

Technical support to RI interpreters varied; some had no support at all, whereas others had solid support from an IT office at the regional county. Several interpreters declared that their needs were so specific that their work separated them from other departments in the organisation. In turn, having such unique needs in an organisation made it difficult to receive help from the regular IT office:

*What often becomes – our unit within the region [is served by] the usual IT guys ... who work well with [all departments]. But what often becomes our problem is that we have such specific requirements and needs compared to everyone else.* (#6)

However, for RI interpreters in other organisations, the IT office was well informed and extremely helpful. In addition, some organisations had their own support for technical problems as well as their own group with interpreters to facilitate RI:

*[A]fter all, we have both this region-wide IT and then we also have an internal IT group that works hard with all technical solutions, [and] also calls around and checks which care units and such are able to take remote assignments.* (#12)

In several cases the interpreters themselves were both supporting and training their colleagues in RI. The interpreters were also assisting the interlocutors with the technical set-up and guiding them on how to use the technical devices. The interpreters had to both solve their own problems and others’ issues related to RI: *[W]e got to be the experts and the guinea pigs at the same time* (#3).

When interpreters were first confronted with RI, their work involved a great deal of trial and error or consulting colleagues in order to master the new situation. Then, interpreters came together, some meeting occasionally and others more regularly – to provide support, training, information, and useful tips to their colleagues. The support was offered individually and to small and larger groups of interpreters. RI issues might also be brought up as a separate point in the workplace meetings. Thus, interpreters exchanged experiences in more or less official ways.

Some organisations provided training sessions for RI. One interpreter described an educational effort offered by the interpreter organisation (Swedish Sign Language Interpreters Community; STTF). In addition, several interpreters mentioned that the video relay training they received during their basic training as interpreters facilitated their work in RI.

To disseminate information among interpreters and to (both signing and speaking) users of RI, material was written down and organised:

*We have acquired manuals that are available on a website and we have a special function mailbox where they [the users of the service] can send in questions and/or problems that have arisen in remote interpreting. We have called interpreter users. At the beginning…, when we had to rearrange all the interpreting assignments, we called the care providers, for example, and explained what [technical equipment] they needed and how it worked, and we also tested [the equipment] with them if they felt unsure. And the same thing with the interpreter users, we explained how it would work and so on.* (#10)

In some cases, the training focused mainly on how to use the technology and did not include other, non-technical, aspects of working in RI:

*I don’t know if they have gone into the soft values ​​so much, like the order of the meeting, what to think about and so on, I don’t think so, although I don’t know, but I have mostly heard about the technology.* (#14)

#### RI is a work in progress, but is here to stay

RI was kickstarted due to COVID-19. Some interpreting services had included some kinds of remote assignments before, but the pandemic forced the evolution:

*We were thrown into it all at once, it wasn’t a project that was one, two, three, four months in the future that lurched along at its leisurely pace, but in one week, all of a sudden, everyone started interpreting remotely.* (#13)

The remote assignments were thus a relatively new and unexplored way to interpret, but they were quickly set up. As time went on, the agencies made improvements and the service got better and better:

*[W]e also created an evaluation document which we sent along, asked them [users of the service] to evaluate both the technology [and] the interpretation. Yes, there were a few different things where we asked how we could improve, and [that was] quite early in the pandemic.* (#23)

The transition to more RI creates possibilities for different regional counties to cooperate. Interpreters that were not used to working together could help each other. This, together with less travel for interpreters, made it possible to carry out more assignments:

*[Y]ou can help each other more between regions. That all of a sudden, the Stockholm region can actually get in touch and say: Now we have a shortage of interpreters this Thursday, could you help us and take these jobs?* (#15)

From the beginning of the pandemic, it was not an option to carry out assignments face-to-face. However, as the pandemic eased, more assignments were done face-to-face. Yet those initial actions taken to address the novel situation caused by COVID-19 are now commonplace for the interpreting agencies. After the pandemic, politicians in one county even specified that a certain proportion of interpretation assignments should be RI:

*The decision is from the regional board, so it is the politicians who have made the decision about 20 percent, so it is in our operational goal. It is a goal that we as a business have no way of influencing.* (#12)

The coming evolution of RI is thus not only an issue for the interpreting agencies, nor does it involve only interpreters’ or users’ preferences; in some places it is an issue well beyond the control of interpreters and users.

## Discussion

The results of the current study about the interpreters’ perceptions of the organisation of RI in Sweden as it has evolved since the COVID-19 pandemic show processes that are unevenly regulated with significant variations across the country. These variations concern regulations, how RI is offered and organised, and working conditions for interpreters. The issue of offering SLI remotely is already found in government documents from 2011 [16], which proposed a single state agency for SLI service, although it was not realised. The analysis of the results in this study shows that this could be a way to go. The regional counties around Sweden could for example cooperate to provide RI. As the results of this study also stress that there is considerable variation across the country, one united agency could jointly review how to organise RI so that it is organised in a uniform way. A uniform RI service which could provide the service in a way that is more equitable across regional boundaries is necessary. As a comparison, the police forces in Sweden were organised locally and regionally but has been transferred to state control. This shows that the transfer of organisation can create a more unified and centralised governance of important societal functions. The same could be achieved for RI services.

Results from the current study could be seen as revealing a lack of official stringency (cf. [24]), whereas the interpreters found solutions on an individual basis. A centralised and well-established RI service could strengthen users’ equal access and options. An option to freely choose between meeting face-to-face or remotely could empower people, giving them greater control over their lives [25]. The control and power could be transferred from the institution providing the service to the users of the service [26]. In the current situation, people who use interpreter services cannot always choose the type of interpretation service they prefer to use. To achieve the same degree of access to the society as others have, the possibility to choose between RI and face-to-face could be one path to take.

The interpreting agency is designed to meet users’ needs. Users of the services provided by regional counties are mainly “childhood deaf, deafblind, adult deaf and hard of hearing” [9]. In the results of this study, it appears that only one agency has had a consulting workshop with the user council; consultations with user councils were not mentioned by any other informants in the study. This does not mean that there have not been discussions with user councils. A drawback from the 2003 directive [13] is that it lacks users’ perspectives and research on SLI. In addition, in the government document from 2011, it was stated that RI should take users’ perspectives and needs into account ([1], p. 23). The service users’ involvement could be developed, and a framework could strengthen sustainability, which could be beneficial for the services (cf. [27]). It is essential to consider the preferences and thoughts of the users of RI, and how we can avoid the same failure to consult those who use interpreting service.

In addition, although the service is provided for its users [9], RI needs to be feasible, and the working conditions for the interpreters have to be appropriate (cf. [6]). Different regional counties and agencies provided very different levels of support in the form of adjustments for interpreters working remotely. A uniform service and equal conditions could have a sustainable impact on interpreters’ everyday work as well as keeping them within the profession.

During the transition to RI due to COVID-19 [2, 6, 28–31], one might ask how all the new experiences have been considered and have impacted the organisation – what is left of the discussion of RI and what will happen next. A persistent issue concerns training and education, which is lacking according to our results. Therefore, consideration should be given to how a formal RI education programme should be organised. The issue also corresponds to efforts to achieve sustainability and excellence in RI. Thus, there is a need for comprehensive training programmes to address both the technical aspects of RI and the interpersonal skills. In addition, there are several stakeholders and interests involved in the issue of organisation of RI. Another question for future research is how (for example) politicians’ decisions, interpreter agencies, users’ needs, interpreters’ needs, and equality should be approached in a democratic and sustainable manner.

### Strengths and limitations

This study is based on interpreters’ comments on the organisation of RI in their regional counties. This is both a strength and a limitation. A strength is that we received an unfiltered situational description of reality. From a management perspective, the informants’ observations reflect what permeates the organisation as experienced by those who work in the service. On the other hand, the interpreters might be unaware of all the decisions and consultations that have taken place at higher levels in the organisation. What can be concluded, though, is that RI, in accordance with SLI from the 2022 investigation, is “still immature and in the early stages of its development” ([1], p. 350).

Another strength is that the authors approach the study from different angles. While the first author (CW) is a certified interpreter who has worked and done research on RI earlier, the second author (MM) is an associate professor in social work and has worked with council organisations before, although not with interpreting agencies per se. This provided an opportunity to generate analysis both from an insider perspective, with a deeper knowledge, and a complementary outsider perspective. In the first step, the interviews were conducted with a deep knowledge of interpreters’ everyday work. In the next step, the first analysis was done by the second author from the outsider perspective.

## Conclusions

This investigation of RI organisation in Sweden as it has evolved since the COVID-19 pandemic has shown that RI is still a work in progress. The regulation of RI varied greatly among regional counties in Sweden with respect to directives, decisions, and platforms used. A great variety of assignments were carried out remotely, and the restrictions varied as interpreter services adapted to the COVID-19 work environment. The risk of making a transition from face-to-face interpreting to RI was taken into consideration in some regional counties, whereas others did not do so in a formal way.

Modifications of interaction were, to some extent, adjusted to user preferences and capabilities. According to interpreters, work environments and workplaces were adjusted, both by rebuilding studios and by adapting spaces to suit furniture and technical devices.

The overall evolution followed the same patterns as the other aspects of organisation: support and training varied, with some having their own technical support and others required to solve their own problems. Common problems were often overcome by cooperating and exchanging helpful tips within the interpreting group.

With unclear regulations and lack of training opportunities, the interpreters themselves seemed to be dealing with the rather new situation as well as they could. It is not clear how RI will evolve over time. In some cases, the issue is outside of the control of interpreters and users, and even of the regional counties. But it is certain that RI in some form is here to stay, as *one* way to receive SLI, and more uniform regulations and a formal training programme would surely have a positive impact on its evolution.

## Supplementary Information

Supplementary Material 1.

Supplementary Material 2.

Supplementary Material 3.

## Data Availability

The datasets used and/or analyzed during the current study are available from the corresponding author on reasonable request.

## Abbreviations

RI: Remote Interpreting
SLI: Sign Language Interpreting
STS: Swedish Sign Language
