# Supplementary material for: Regulation, modification, and evolution of remote sign language interpreting in Sweden – a service in progress
Source: BMC Health Serv Res. 2024 Nov 19;24:1431. doi: 10.1186/s12913-024-11907-y (PMC11575209; doi:10.1186/s12913-024-11907-y)
Supplement: Supplementary file 1 — Supplementary Material 1. [file 12913_2024_11907_MOESM1_ESM.pdf]

Semi-structured interview guide

- What determines whether an assignment becomes RI or face-to-face (f-t-f) assignment?
- What types of assignments are/are RI assignments?
- Are there situations when RI cannot be used?
- Do the same rules apply in RI as on site?
- Where is RI performed (at home or another location)?
- How is confidentiality guaranteed (in case of possible work in a home environment)?
- What types of technical solutions have you used when you have interpreted remotely?
- Who has decided which technical solution was used?
- Is there anything organizational that would facilitate your work in RI?
- Has the workplace been adapted to RI based on Covid-19 in any way?
- How do you experience the physical work environment during RI compared to f-t-f?
- How do you experience the psychosocial work environment when interpreting remotely?
- What training/practice have you received to be able to perform RI?
