# Supplementary material for: Regulation, modification, and evolution of remote sign language interpreting in Sweden – a service in progress
Source: BMC Health Serv Res. 2024 Nov 19;24:1431. doi: 10.1186/s12913-024-11907-y (PMC11575209; doi:10.1186/s12913-024-11907-y)
Supplement: Supplementary file 2 — Supplementary Material 2. [file 12913_2024_11907_MOESM2_ESM.pdf]

Content to participate in the study

I have received oral/sign language and written information about the study and have had the opportunity to ask questions. I keep the written information.

☐ I agree to participate in the study Perspectives of remote interpreting between a spoken and signed language.

☐ I agree to data about me being processed in the manner described in the research subject information.

Place and date	Signature
	Name
