# Supplementary material for: Regulation, modification, and evolution of remote sign language interpreting in Sweden – a service in progress
Source: BMC Health Serv Res. 2024 Nov 19;24:1431. doi: 10.1186/s12913-024-11907-y (PMC11575209; doi:10.1186/s12913-024-11907-y)
Supplement: Supplementary file 3 — Supplementary Material 3. [file 12913_2024_11907_MOESM3_ESM.pdf]

Samtycke till att delta i studien

Jag har fått muntlig/ teckenspråkig och skriftlig informationen om studien och har haft möjlighet att ställa frågor. Jag får behålla den skriftliga informationen.

☐ Jag samtycker till att delta i studien Perspektiv av tolkning distans mellan talat och tecknat språk

☐ Jag samtycker till att uppgifter om mig behandlas på det sätt som beskrivs i forskningspersonsinformationen.

Plats och datum	Underskrift
	Namnförtydligande
